# A Trade-Off between Reproduction and Feather Growth in the Barn Swallow (*Hirundo rustica*)

**DOI:** 10.1371/journal.pone.0096428

**Published:** 2014-05-14

**Authors:** Nicola Saino, Maria Romano, Diego Rubolini, Roberto Ambrosini, Andrea Romano, Manuela Caprioli, Alessandra Costanzo, Gaia Bazzi

**Affiliations:** 1 Department of Biosciences, University of Milan, Milan, Italy; 2 Department of Biotechnology and Biosciences, University of Milano-Bicocca, Milan, Italy; University of Lausanne, Switzerland

## Abstract

Physiological trade-offs mediated by limiting energy, resources or time constrain the simultaneous expression of major functions and can lead to the evolution of temporal separation between demanding activities. In birds, plumage renewal is a demanding activity, which accomplishes fundamental functions, such as allowing thermal insulation, aerodynamics and socio-sexual signaling. Feather renewal is a very expensive and disabling process, and molt is often partitioned from breeding and migration. However, trade-offs between feather renewal and breeding have been only sparsely studied. In barn swallows (*Hirundo rustica*) breeding in Italy and undergoing molt during wintering in sub-Saharan Africa, we studied this trade-off by removing a tail feather from a large sample of individuals and analyzing growth bar width, reflecting feather growth rate, and length of the growing replacement feather in relation to the stage in the breeding cycle at removal and clutch size. Growth bar width of females and length of the growing replacement feather of both sexes were smaller when the original feather had been removed after clutch initiation. Importantly, in females both growth bar width and replacement feather length were negatively predicted by clutch size, and more strongly so for large clutches and when feather removal occurred immediately after clutch completion. Hence, we found strong, coherent evidence for a trade-off between reproduction, and laying effort in particular, and the ability to generate new feathers. These results support the hypothesis that the derived condition of molting during wintering in long-distance migrants is maintained by the costs of overlapping breeding and molt.

## Introduction

The simultaneous expression of major organismal functions is often constrained by limiting energy, material resources or time [1] [2]. Physiological trade-offs among competing activities such as reproduction and self-maintenance are a major force shaping the evolution of life-histories, including annual routines, i.e. the temporal organization of activities over the annual cycle [3] [4].

In birds, plumage renewal, either in the form of molt or of replacement of feathers which are accidentally lost, is an essential activity to retain efficient aerodynamics, thermal insulation and sexual signaling, and is one of the most energy- and time-consuming activities [5], [6], [7], [8], [9], [10], [11]. Feathers consist almost exclusively of proteins rich in limiting dietary sulfur amino acids, and may build up to 40% of the total dry, lean mass of a bird [6], [7], [12], [13]. Because of its energetic costs, feather biosynthesis may thus to have to be traded against allocation to reproduction, which is also highly demanding [14], [15], [16], [17], [18], [19].

In fact, the diverse molt strategies that birds have evolved [12], [20], [21] may reflect selection for optimization of annual routines by reducing the impact of any trade-offs arising from overlap of molt with reproduction and migration [5], [22], [23], [24], [25]. Partitioning of molt from reproduction and migration is the prevailing strategy, although partial overlap may occur when time is constraining annual cycle and is more frequent in the tropics [26], [27], [28]. In species breeding in temperate boreal latitudes, summer molt appears to be the ancestral state, while winter molt is a derived state that has evolved in species that winter south of the Sahara [29], [30]. Winter molt in these species possibly results from tightness of annual routines, whereby early onset of autumn migration prevents post-breeding molt, and/or from conditions in the winter quarters favoring this alternative molt strategy (e.g. [31]). In addition to periodic partial or complete molt, however, birds can replace feathers that may be accidentally lost [32], [33], suggesting strong selection for maintenance of aerodynamic and insulatory plumage integrity.

The costs of reproduction, and the adaptations to sustain such costs have been at the focus of a huge number of studies. Conversely, current knowledge on the costs of feather renewal is still sparse. Specifically, empirical evidence from field studies on the trade-off between reproduction and the ability to grow new feathers, which may select for maintenance of temporal disjunction between breeding and molt which is observed in so many species, is scant (e.g. [15], [17], [18], [34], [35], see also [36]).

In the present study we experimentally investigated the trade-off between feather production and breeding in the barn swallow (*Hirundo rustica*), a long-distance migratory passerine whose European breeding populations undergo a single annual molt of the tail and wing feathers during wintering (October – March) in sub-Saharan Africa [37], [38]. We removed one tail feather from a large sample of adults and analyzed the growth of the replacement feather in relation to stage in the breeding cycle when the feather had been removed and to reproductive effort, as gauged by clutch size. As indicators of the ability to replace the feather we measured the length of the replacement feather before growth of the replacement feather had been completed and the width of the growth bars (see below). This dual approach was adopted because the ability to replace a feather depends both on latency between feather removal and the start of growth of the replacement feather, and on the rate of growth of the new feather, as reflected by the width of growth bars.

Feather growth outside the normal molting period does not reflect ‘true’ molt, which would be prohibitively difficult to monitor at the individual level during wintering of free-ranging long-distance migrants in Africa. However, the approach of studying growth of a replacement feather produced during the breeding season that we adopted here provides information on feather regeneration ability in relation to breeding stage and reproductive effort. This information can shed light on the selection pressures that prevent the evolution of overlap between molt and breeding in species which currently show obligate segregation between these activities.

Growth bars are regular successions of light and dark bands perpendicular to the rachis, a few millimeters in width, that several species display mostly on their rectrices and remiges [39], [40], [41], [42], [43]. The width of the growth bars (GBW) is considered a proxy for feather growth rate, as a pair of consecutive light/dark bands reflects a 1-day growth interval [39], [44], [45], [46], [47], see also [48]; but see [49] [50]). Thus, wider growth bars reflect faster feather growth. Such ptilochronological variable is known to covary with general state and condition of the bird at the time when the feather was produced, as well as to depend on several extrinsic factors (e.g. [51], see references in [42]). Condition-dependence of GBW (e.g. [44], [52], [53]) implies that they can serve as useful tools in the study of physiological trade-offs.

If feather production is traded against breeding, we expected GBW and length of the growing replacement feather at any given time after removal of the original feather to decline from the pre-laying to the laying and incubation/nestling period for females, because of allocation of resources to egg production, of time devoted to incubation rather than to foraging, and to food provisioning of the nestlings. We had no unequivocal predictions for males because the change in the costs of socio-sexual behavior during the breeding cycle relative to parental behavior are poorly known. However, if feather growth is traded against breeding, we expected GBW on replacement feathers and also their length at growth completion to be smaller than that of the homologous feather grown during the normal molt period.

Importantly, to test for a trade-off between the amount of resources allocated to reproduction and the ability to grow new feathers, we also analyzed the covariation between the number of eggs laid and GBW or length of the growing replacement feather in females, while predicting a negative relationship, possibly more strong for females with large as compared to small clutches and for females that had to replace the feather during or after clutch completion rather than before laying. We did not expect any such relationship for males.

## Methods

### Ethics Statement

Upon capture, barn swallows were kept in cloth bags in a safe position, as is standard practice in bird ringing studies. One tail feather was removed by gently pulling the feather from the distal end. All individuals were released as soon as possible, usually within 1 hour of capture. After being released, swallows behaved normally and observations at the nest on dozens of individuals confirmed that they resumed their normal breeding activities. The study was carried out under permission of the local authority (Provincia di Novara #4309/2011) responsible for authorizing animal studies in the wild. The farmers gave permission to enter their properties. No approval from an ethical committee was required for this study.

### Model Organism

The barn swallow is a semicolonial, long-distance migratory passerine which forages on the wing on flying insects. European breeding populations winter in Sub-Saharan Africa [37], [38]. Socially monogamous pairs have up to three clutches of 2–7 eggs (modal clutch size is 5 eggs), laid at one-day intervals. In the European populations, females alone incubate the eggs, for ca. 14 days. Both parents attend the offspring that fledge 18–21 days after hatching.

### Field Procedures

During spring-summer 2012 and 2013 we studied barn swallows at 16 colonies ( =  farms) in Piemonte (center of the area: 45°33′ N, 8°44′ E) and Lombardia (45°19′N, 9°40′E), in Northern Italy. The nests inside cowsheds and other buildings were visited at regular intervals (2–10 days) to record date of first clutch initiation (see Supporting Information S1). In up to 4 sessions over the breeding season (April – July), all adults were captured using mist nests, sexed and colour-ringed for later assignment to their nest by observation. At first or second capture, the 4^th^ (counting outwards) right rectrix feather (OrR_4_) was plucked and stored in a plastic bag. After 24–63 days we recaptured the adults and removed the replacement feather (ReR_4_) in order to measure the length and growth bar width on it. Approximately 91% of the recaptures occurred between day 24 and day 35 after plucking because we aimed at measuring the length of the growing feather as an indicator of feather regeneration ability, rather than length at growth completion. Thus, the ReR_4_ was removed and its length was measured on different individuals at different times since removal of the OrR_4_. This was the case because recapturing hundreds of individuals exactly at the same time since OrR_4_ removal was impractical. We therefore corrected ReR_4_ length measurements for time since OrR_4_ removal (see *Statistical analyses*). It should be noted that length of growing ReR_4_ depends both on the time taken by the ReR_4_ to start growing and on its growth rate (see SI.2). The remaining 9.3% recaptures occurred between day 40 and day 63 after OrR_4_ removal, when growth had been completed (see SI.2).

### Growth Bar Width and R_4_ Length Measurements

We measured GBW of the OrR_4_ and ReR_4_ because previous observations (see [42]) showed that OrR_4_ is the rectrix or remex feather where bands can be identified most clearly. The birds (10%) where too few or no distinct growth bars could be distinguished were excluded. To estimate GBW, on the dorsal surface of the vane we identified the proximal and distal limits of a feather segment including 9 growth bars. The segment started from the second clearly visible bar at the distal end of the feather. The limits of the segment where marked on the rachis with a fine white fibre tip pen. The length of the segment was measured with a digital caliper (precision of 0.01 mm) under a light-emitting diode in a semi-obscure chamber (see SI.3). GBW was expressed as the length of the segment/9 (see [42], [43], [44], [54]). Hence, large GBW indicates rapid feather growth. Repeatability of GBW measures on R_4_ and correlation between GBW on rectrices and remiges are high [42], [43].

Length of the removed OrR_4_ or the ReR_4_ was measured as the distance from the inferior umbilicus to the distal end of the vane on scans of the feathers, where we included a ruler as a reference, by ImageJ 1.46r program. Using the “Segmented line” tool we could account for slightly curved shape of the calamus and of the rachis.

### Statistical Analyses

We used linear mixed models to analyze GBW on ReR_4_ in relation to breeding stage at OrR_4_ removal, date at OrR_4_ removal (herafter “date”), and clutch size (i.e. number of eggs at clutch completion) and sex (factor). Breeding stage was expressed as the difference in days between the date of removal of OrR_4_ and the date of laying of the first egg of the first clutch by the female of the pair to which the individual belonged. Breeding stages ranged between −20 and+30, i.e. between 20 days before laying of the first egg of the first clutch and the mid-late nestling stage of the first brood. Thus, all replacement feathers were grown during the first breeding attempt. First and second-order polynomial terms of breeding stage and clutch size were initially included in the models. Polynomial multiple regression models were simplified by step-down removal of the non-significant terms. Because the study was carried out in two years and individuals were clustered in colonies, in the mixed models we initially also entered colony or year as random effects and compared the fit of these models with that of a null (random-intercept) model by Likelihood-Ratio tests. In the analyses of GBW, the random effect of year was found to significantly increase the fit of the model and year was therefore retained in the random effects specifications of the model (LR tests; *p*<0.05 in all cases). Conversely, colony was never found to significantly improve the fit of the mixed models (*p*>0.05) and was therefore always excluded from the models. A small fraction (15% of the males; 13% of the females) was included in the sample of both years. Because the proportion of birds sampled in both years was small and preliminary linear mixed model analyses showed that inclusion of individual as a random factor did not significantly improve the fit of the models (LR tests; *p*>0.05 in all cases), for simplicity we treated the repeated observations from the two years as independent. In the analyses of the residuals of the length of the growing ReR_4_ (see below), the random effects of year, colony and individual did never significantly contribute to the fit of the models (LR tests; *p*>0.05 in all cases). These analyses were therefore run as linear models with fixed effects only.

Piecewise regression analysis was used to identify any discontinuity in variation of GBW or growing ReR_4_ length in relation to breeding stage after tentatively identifying any discontinuity in variation by means of LOESS regression [55]. When the piecewise regression algorithm failed to converge, we relied on polynomial regression to identify non-linear trends and maxima/minima (see SI.4).

The analyses of the length of the growing ReR_4_ data were based on the ratios between ReR_4_ length at measurement and length of the OrR_4_. The approach of using relative as compared to absolute lengths is better than using absolute ReR_4_ length because it automatically accounts for inter-individual variation in tail feather length. The (ReR_4_ length)∶(OrR_4_ length) ratios in relation to time since removal of OrR_4_ were fitted with a Gompertz function for either sex separately. The goodness of fit of the Gompertz models was expressed as *R^2^* = 1-(Residual SS –SS). Residuals of individual ReR_4_ length (hereafter “LengthRe” for consistency and brevity) from the Gompertz regression were computed and used as an index of the actual feather growth. Thus, individuals with large LengthRe were those that, at any time after removal of the OrR_4_, had longer growing replacement feather relative to the other individuals, because of the combined effects of daily rate of feather growth and time elapsed from OrR_4_ removal till the start of ReR_4_ growth (see also above). LengthRe measured before day 40 after plucking of the OrR_4_ (i.e. the day when replacement R_4_ could be assumed to have completed growth; see Results) were analyzed in relation to breeding stage (second-order polynomial terms), clutch size (second-order polynomial terms) and date in linear models. ReR_4_ feathers plucked 40 or more days after OrR_4_ removal were used to compare the absolute lengths of the OrR_4_ and the ReR_4_.

To explore variation in the association between GBW or LengthRe and clutch size we restricted the analyses in turn to all possible ranges of breeding stages at OrR_4_ removal varying in amplitude between 9 days and 51 days (i.e. the maximum span of breeding stage between −20 and +30), and differing in duration by multiple of 3 days for the analyses with LengthRe, or between 20 and 50 days and differing in duration by multiple of 10 days for the analyses with GBW (see SI.5 for a full explanation of this procedure). Variation in the strength of the association with LengthRe was summarized by contouring the *t*-values for the effect of clutch size from multiple regression models in a triangular biplot with median breeding stage of the particular interval on the *x*-axis and duration of the interval on the *y*-axis.

The analyses were run with SAS 9.2, SPSS13 and Surfer 7.0 statistical packages.

## Results

### Growth Bar Width (GBW) of the Replacement Feather

We measured GBW of the ReR_4_ in a sample of 188 males and of 188 females whose OrR_4_ had been removed. The median number of birds sampled in the 16 study colonies was 24 (range: 4–54 individuals). The number of birds sampled was 207 in 2012 and 169 in 2013. Mean breeding stage at OrR_4_ removal relative to first clutch initiation was −1.8 days (SD±10.5) for males and −0.1 days (SD±10.4) for females.

**Figure 1:**
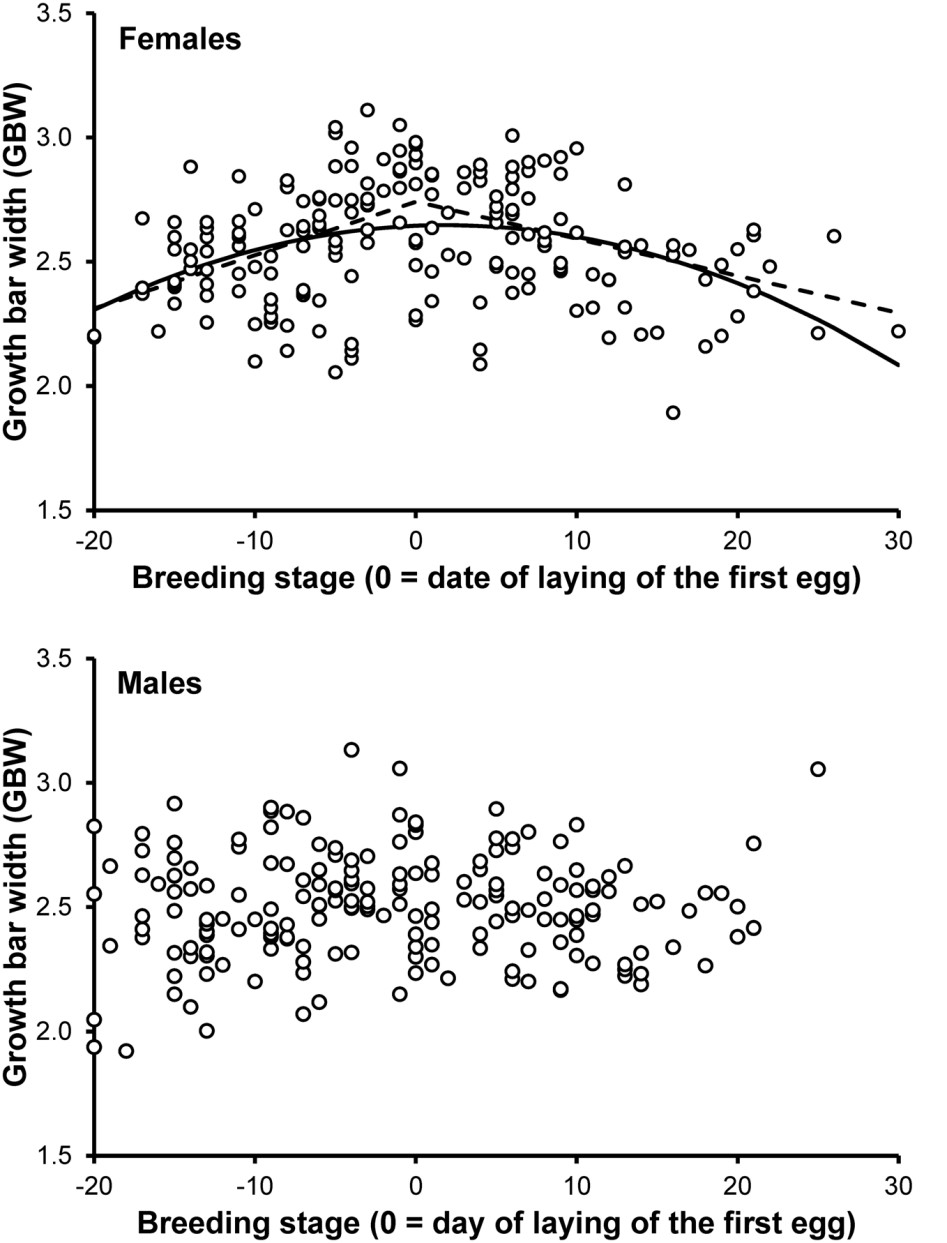
Growth bar width in relation to breeding stage. Growth bar width of the replacement R_4_ in relation to breeding stage when the original R_4_ had been removed. For females, the two functions fitted by piecewise regression analysis and the curvilinear function fitted by a linear mixed model with second-order polynomial terms of breeding stage are shown.

We first explored variation in GBW using LOESS regression on either sex separately. Visual inspection of LOESS curves suggested that in both sexes a discontinuity existed around the day of clutch initiation (breeding stage = 0) by the female of the pair. In females, piecewise regressions on either sex separately using breeding stage = 0 as a tentative breakpoint provided a highly significant fit of the data (*F*_3,184_ = 13.75, *p*<0.001; Model Standard Error = 0.049; breakpoint estimated by the model at *x*  =  −0.998 (CI: −4.120–2.125)) (Fig. 1). The regression coefficient for *x* values before the breakpoint was positive and its confidence interval did not include 0 (estimate = 0.022; CI: 0.012–0.031). After the breakpoint, the slope was negative and its confidence interval again did not include 0 (−0.015; −0.022– −0.008).

A mixed model with second order polynomial terms of breeding stage and date of OrR_4_ removal as fixed effects revealed an inverse U-shaped pattern of variation (breeding stage: F_1,184_ = 1.73, p = 0.190, coefficient: 2.3×10^−3^ (1.7×10^−3^); breeding stage^2^: *F*_1,184_ = 36.82, *p*<0.001, coefficient: −7.3×10^−4^ (1.2×10^−4^); date of OrR_4_ removal: F_1,184_ = 1.31, p = 0.255, coefficient: 2.0×10^−3^ (1.7×10^−3^)) (Fig. 1). Thus, polynomial regression indicated that a maximum in GBW was attained at breeding stage = 1.5. Hence, polynomial and piecewise regressions consistently indicated a maximum in GBW of females to occur around clutch initiation. In the remainder of the analyses of GBW we therefore used breeding stage ≤ 0 as a cutoff.

On the other hand, piecewise regression on male data failed to provide a significant fit of the GBW data (*F*_3,184_ = 1.54, *p* = 0.205). Similarly, a linear mixed model with second order polynomial terms of breeding stage as predictors did not show any significant curvilinear variation of GBW (*F*_2,185_ = 1.27, *p* = 0.282) (Fig. 1). In addition, no linear variation of GBW was found in a linear mixed model with breeding stage (*F*_1,185_ = 1.79, *p* = 0.182) and date of OrR_4_ removal (*F*_1,185_ = 0.67, *p* = 0.415).

A linear mixed model with sex as a factor indicated that GBW did not differentially change with breeding stage in either sex for breeding stages ≤ 0, although the sex by breeding stage effects was marginally non-significant (Table 1). Conversely, the relationship between GBW and breeding stage differed between sexes for breeding stage values>0 (Table 1).

**Table 1:** GBW of the replacement feather in relation to stage in the breeding cycle.

		*F*	d.f.	*P*		Coefficient (±SE)
Linear regression: breedingstage≤0						
	Sex	6.04	1, 217	0.015		
	Breeding stage	21.90	1, 217	<0.001		
	Sex x Breeding stage	3.78	1, 217	0.053	Males	
	Feather removaldate	1.31	1, 217	0.253		
Linear regression: breedingstage>0						
	Sex	13.80	1, 149	<0.001		
	Breeding stage	8.54	1, 149	0.004		
	Sex x Breedingstage	11.27	1, 149	0.001	Males	8.8×10^−4^ (4.0×10^−3^)
					Females	−1.6×10^−2^ (3.2×10^−3^)
	Feather removaldate	0.83	1, 149	0.365		
Polynomial regression						
	Sex	13.9149	1, 369	<0.001		
	Breeding stage	3.416	1, 369	0.066		
	Breeding stage^2^	27.74	1, 369	<0.001		
	Sex x Breeding stage	0.12	1, 369	0.73006		
	Sex x Breeding stage^2^	10.25	1, 369	0.002		
	Feather removal date	1.59	1, 369	0.209		

Linear mixed models of GBW of the replacement R_4_ on breeding stage in the pre-laying (breeding stage ≤ 0) or the post-clutch initiation period (breeding stage>0), and linear mixed model with second order polynomial terms of breeding stage testing for a difference in the relationship between GBW and breeding stage in either sex.

GBW of the ReR_4_ was significantly smaller than GBW measured on the OrR_4_ in both sexes (Table 2), with a larger proportional difference in males compared to females. Overall, GBW of OrR_4_ was slightly larger in males than in females (*t*_365_ = 2.39, *p* = 0.017) whereas GBW of ReR_4_ was significantly larger in females than in males (*t*_374_ = 2.70, *p* = 0.007) (Table 2).

**Table 2:** Mean (±SE) GBW of the original and the replacement R_4_ of either sex.

	GBW_Original_	GBW_Replacement_	Δ%	*t*	d.f.	*P*
Females	2.81 (0.019)	2.57 (0.018)	−8.54	10.51	183	<0.001
Males	2.87 (0.016)	2.50 (0.016)	−12.90	18.22	182	<0.001

The percentage difference in GBW between the replacement and the original R_4_, and the results of a paired *t*-test of the difference are given.

### Length of the Replacement Feather

**Figure 2:**
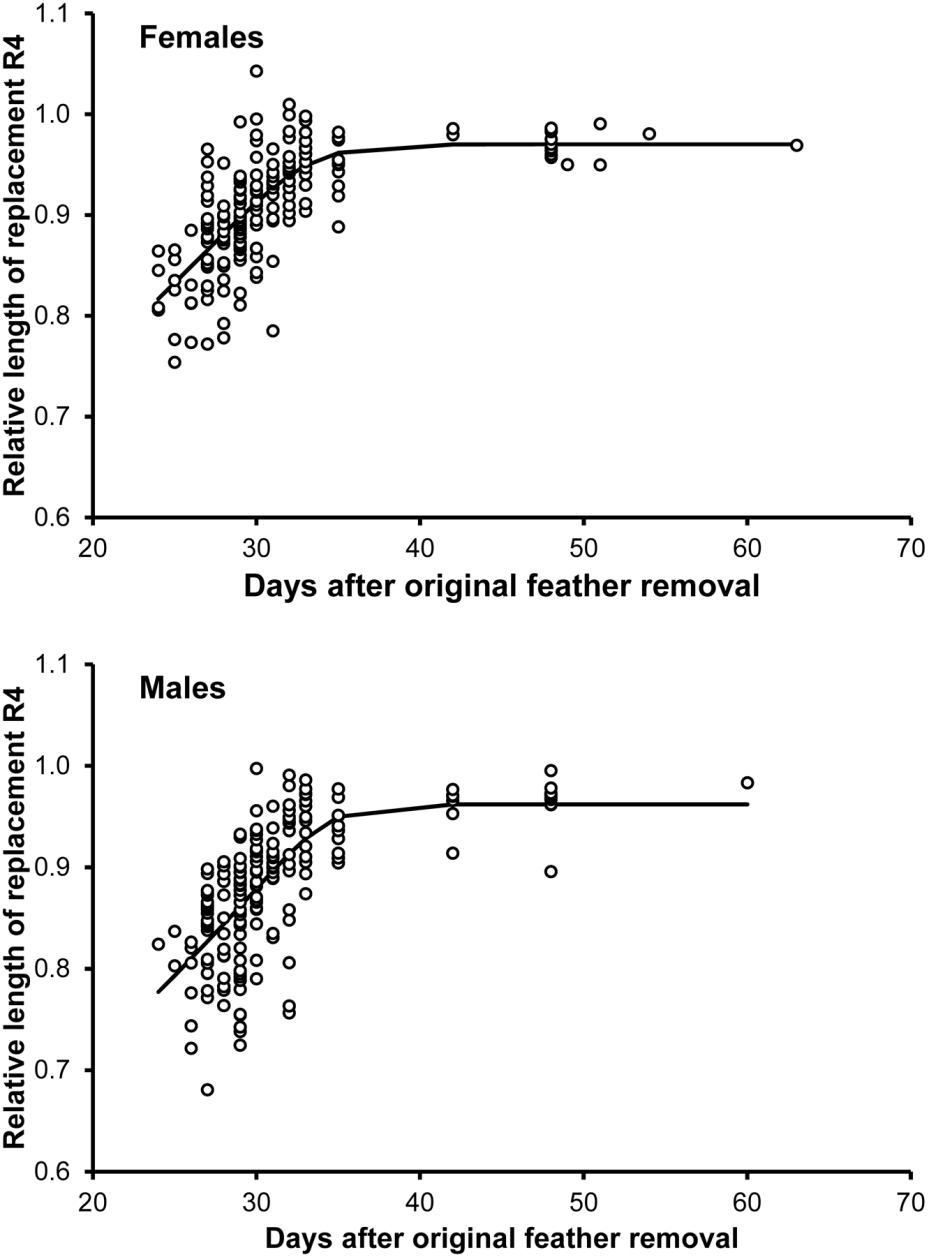
Length of the growing R_4_ in relation to time since feather removal. Length of the growing R_4_ relative to the length of the original R_4_ in relation to time since removal of the original R_4_. The continuous lines represent the Gompertz functions fitted to the data.

**Figure 3:**
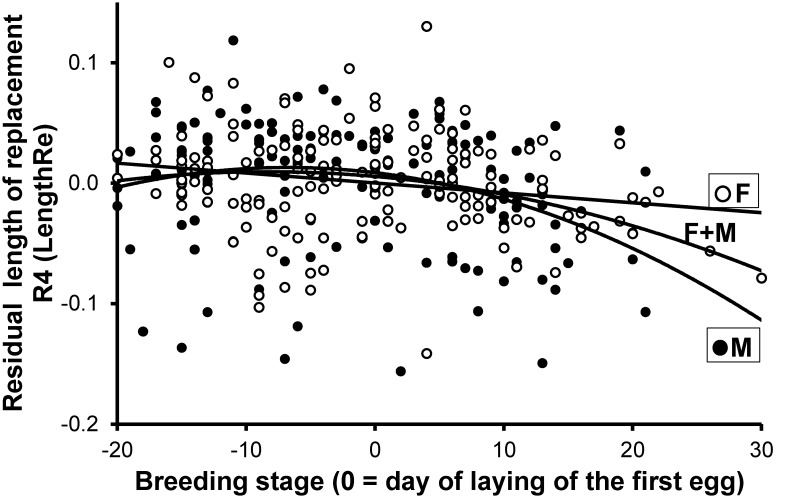
Length of the growing R_4_ in relation to time breeding stage at feather removal. Variation of LengthRe (see Methods) in relation to breeding stage for the replacement feathers measured less than 40 days after removal of the original feather. The linear function fitted to females (F), the second-order polynomial function fitted to males (M), and the second-order polynomial function fitted to the two sexes pooled (F+M) are shown.

Length data of growing ReR_4_ relative to the length of OrR_4_ were subjected to a regression analysis with a Gompertz model in relation to time elapsed since OrR_4_ removal in either sex separately. The fitted Gompertz functions (*R^2^* for males: 0.474; females: 0.510) suggested that ReR_4_ growth was completed by day 40 after OrR_4_ removal in both sexes (Fig. 2). The residuals from the Gompertz regression (LengthRe; see *Statistical analyses*) for males were significantly predicted by the second-order polynomial terms of breeding stage (Table 3). In this model, date positively predicted LengthRe (Table 3, Fig. 3). In females, the quadratic term did not significantly contribute to the model explaining LengthRe (Table 3, Fig. 3). A model excluding this term showed a negative effect of breeding stage and a positive effect of date (Table 3).

**Table 3:** Length of the growing replacement feather in relation to time since removal.

		*t*	d.f.	*P*	Coefficient (±SE)
Females					
	Intercept				−0.156 (0.035)
	Breeding stage	−4.02	153	<0.001	−1.3×10^−3^ (3.1×10^−4^)
	Breeding stage^2^	(−1.65	152	0.101)	
	Feather removal date	4.49	153	<0.001	1.2×10^−3^ (2.6×10^−4^)
Males					
	Intercept				0.166 (0.056)
	Breeding stage	−3.75	153	<0.001	1.4×10^−3^ (3.7×10^−4^)
	Breeding stage^2^	−2.66	153	0.009	−9.3×10^−5^ (3.5×10^−5^)
	Feather removal date	3.15	153	0.002	1.4×10^−3^ (4.2×10^−4^)
Two sexes					
	Intercept				−0.161 (0.031)
	Sex	0.41	308	0.682	
	Breeding stage	−5.08	308	<0.001	1.2×10^−3^ (2.4×10^−4^)
	Breeding stage^2^	−2.98	308	0.003	6.0×10^−5^ (2.0×10^−5^)
	Feather removal date	5.43	308	<0.001	1.3×10^−3^ (2.3×10^−4^)
	Sex x Breeding stage	(−0.51	305	0.608)	
	Sex x Breeding stage^2^	(−1.31	305	0.191)	
	Sex x Feather removal date	(0.33	305	0.743)	

Linear models of the residuals of replacement R_4_ length relative to original R_4_ length on time since original feather removal (LengthRe in the text), on date and breeding stage for either sex separately or for the two sexes pooled. The statistics for the intercept and the first-order term for females are obtained from a model excluding the non-significant second-order polynomial term (in parentheses). Only re-growing feathers measured less than 40 days after plucking are considered (see Fig. 2).

However, a linear model of LengthRe from both sexes failed to disclose significant interactions between sex and the polynomial terms of breeding stage or feather removal date (*p*>0.190; Table 3), which were removed from the model en bloc. The reduced model showed a significant inverse U-shaped variation of LengthRe according to breeding stage and confirmed the positive association with date that emerged from the analyses on either sex separately (Table 3) (Fig. 3). The partial derivative of the fitted function with respect to breeding stage indicates that the maximum was attained at breeding stage −10.3 (see Fig. 3). A model with sex, date and the linear term of breeding stage showed no significant effect of breeding stage on LengthRe (*F*_1,64_ = 0.22, *p* = 0.639) for breeding stages ≤ −11. Conversely, the relationship was highly significant and negative for breeding stages ≥ −10 (*F*_1,241_ = 18.31, *p*<0.001; coefficient = 1.4×10^−3^ (3.3×10^−4^)). Hence, LengthRe did not vary with breeding stage until −10 days before females started laying, whereas it declined afterwards.

In both sexes, the proportion of the length attained by the fully grown ReR_4_ relative to OrR_4_ length was significantly smaller than 1, as determined in the sample of ReR_4_ collected later than day 40 after OrR_4_ removal (H_0_: relative length = 1; H_1_: relative length ≠ 1; males: relative length = 0.964 (0.006), one-sample *t*_17_ = 6.51, *p*<0.001; females: relative length = 0.973 (0.003), *t*_16_ = 8.58, *p*<0.001). However, there was no significant difference in relative final feather length between males and females (independent samples t-test, *t*_33_ = 1.36, *p* = 0.184).

LengthRe was positively correlated with GBW in both sexes (females: *r* = 0.282, *n* = 156, *p*<0.001; males: *r* = 0.404, *n* = 157, *p*<0.001). However, this relatively strong association could partly be due to a spurious effect of the covariation of both variables with breeding stage (linear and quadratic), date and clutch size (linear and quadratic) (see below). A partial correlation analysis controlling for these variables confirmed a significant, though weaker particularly among females, association (males: *r*_par_ = 0.367, d.f.  = 149, *p*<0.001; females: *r*_par_ = 0.172, d.f.  = 147, *p* = 0.036).

### Trade-off between GBW or LengthRe and Clutch Size

**Figure 4:**
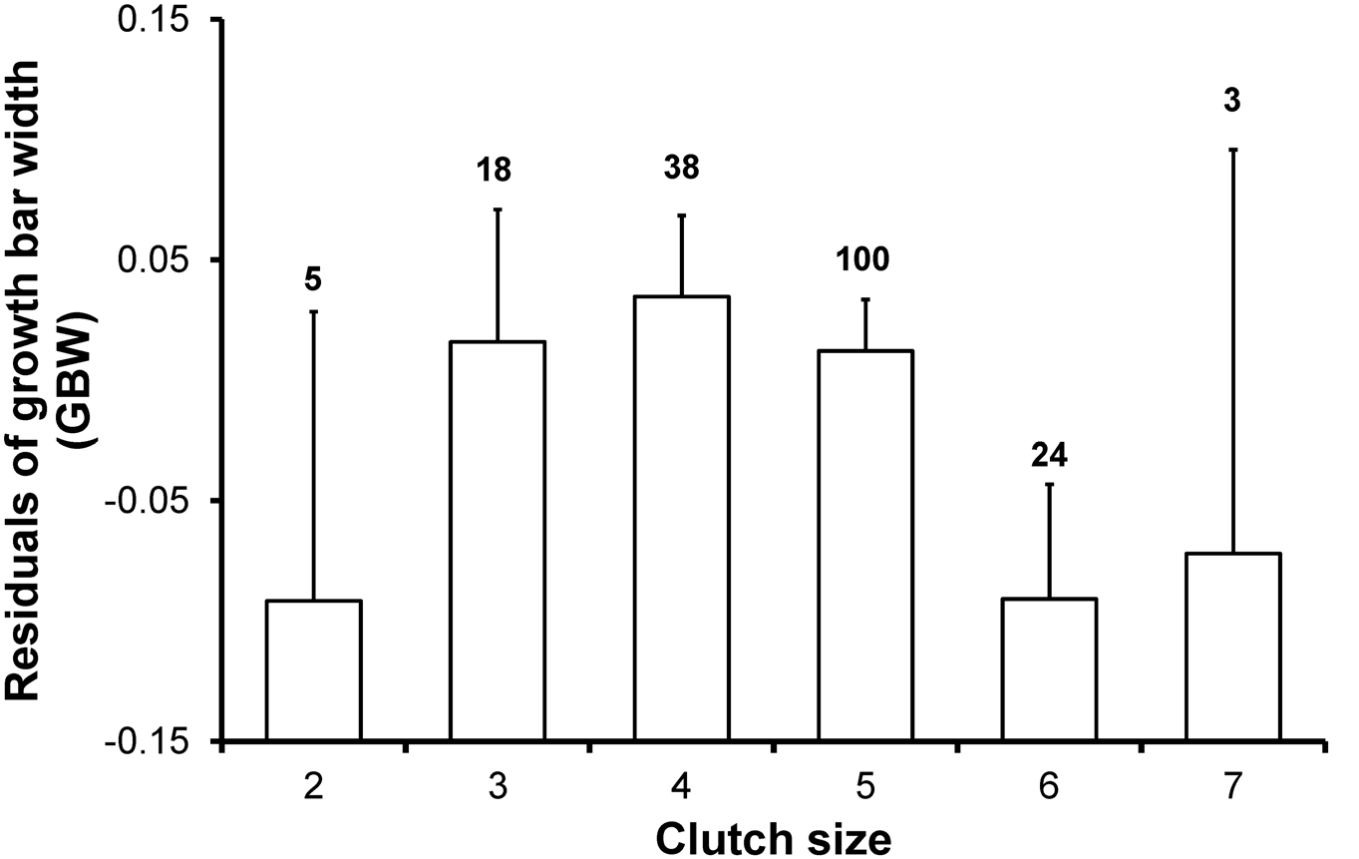
Mean (±SE) residual GBW of females in relation to clutch size. Mean (±SE) residuals of GBW of females from a regression on date and second-order polynomial terms of breeding stage in relation to clutch size. Numbers above bars indicate sample size.

GBW of females was analyzed in relation to clutch size (second-order polynomial terms) in a linear mixed model where we also included breeding stage (second-order polynomial terms) and date. The quadratic term of clutch size weakly and significantly predicted GBW (Table 4, Fig. 4). The partial derivative of the fitted function with respect to clutch size indicated that maximum GBW was attained for clutch size of 3.99 eggs (Table 4; Fig. 4). No significant association of GBW with clutch size existed for clutch sizes<4 (*F*_1,16_ = 0.76, *p* = 0.396) in a model also including breeding stage (second order polynomial terms) and date (other details not shown). Conversely, a model with the same design indicated that GBW significantly declined with clutch size for clutch sizes of 4 or more eggs (*F*_1,160_ = 5.34, *p* = 0.022, coefficient  =  −0.060 (0.026)) (Fig. 4). Linear mixed models restricted to subsets of breeding stages spanning 20, 30 or 40 days and centered at different stages of the breeding cycle (see *Statistical analyses* and SI) revealed significant (0.023<*p*<0.034) associations of GBW with the quadratic term of clutch size in the models spanning over the following ranges of breeding stages: (−11,31); (−1,31); (−21,21); (−11,21); (−1,21). These models showed that a maximum in GBW was always attained for clutch size of ca. 4 (details not shown), as in the model with no restrictions on breeding stage values (Table 4). Thus, GBW seemed to be negatively associated with clutch size only for clutch sizes of 4 or more eggs and throughout the entire range of breeding stages we considered. When the above models were applied to data for males, no hint of any significant association of GBW with clutch size emerged (details not shown).

**Table 4:** GBW of the replacement feather in relation to clutch size.

		*t*	d.f.	*P*	Coefficient (±SE)
Females					
	Intercept				2.110 (0.316)
	Clutch size	2.05	180	0.042	0.210 (0.103)
	Clutch size^2^	−2.28	180	0.024	−0.026 (0.012)
	Breeding stage	1.81	180	0.071	3.2×10^−3^ (1.8×10^−3^)
	Breeding stage^2^	−6.17	180	<0.001	7.5×10^−4^ (1.2×10^−4^)
	Feather removal date	0.64	180	0.524	1.1×10^−3^ (1.7×10^−3^)
Males					
	Intercept				2.073 (0.307)
	Clutch size	0.62	183	0.533	0.049 (0.078)
	Clutch size^2^	−0.59	183	0.555	−5.0×10^−3^ (9.0×10^−3^)
	Feather removal date	1.29	183	0.198	2.4×10^−3^ (1.9×10^−3^)

Linear mixed model of GBW of replacement R_4_ on clutch size, and breeding stage and date at original feather removal. For females, the second-order polynomial term of breeding stage is also included (see Results).

The models in Table 4, in addition, confirmed the inverse U-shaped association of GBW of females with breeding stage (see also Table 1).

**Figure 5:**
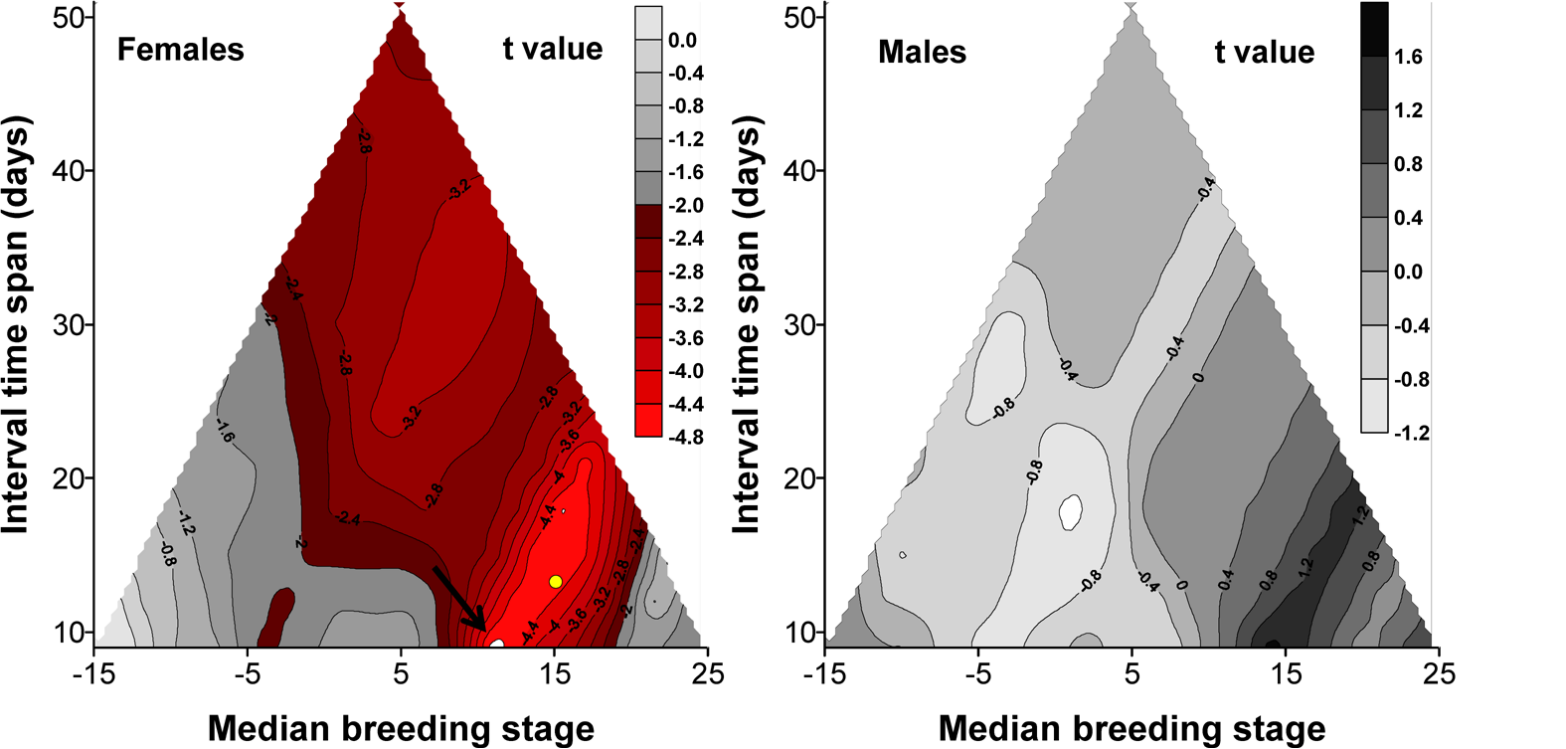
Strength of the association between growing R_4_ length and clutch size during the breeding cycle. Contouring of *t*-values associated to the effect of clutch size on LengthRe (see Methods) of females obtained in multiple regression models also including breeding stage and date at original feather removal (see Table 5). *t*-values were obtained from models including ranges of breeding stages centered at different median values and differing in width (see SI.5). Thus, for example, the yellow dot indicates a model where breeding stages spanning from breeding stage 10 and 21, and centered on breeding stage 15.5 were considered. Red isopletes indicate breeding stage ranges where unsigned *t*-values were significant. The arrow indicates the range where the unsigned *t*-value was largest. The apex of the triangle denotes the t-value of the models including the entire range of breeding stages (i.e. all data points) (see Table 5).

**Figure 6:**
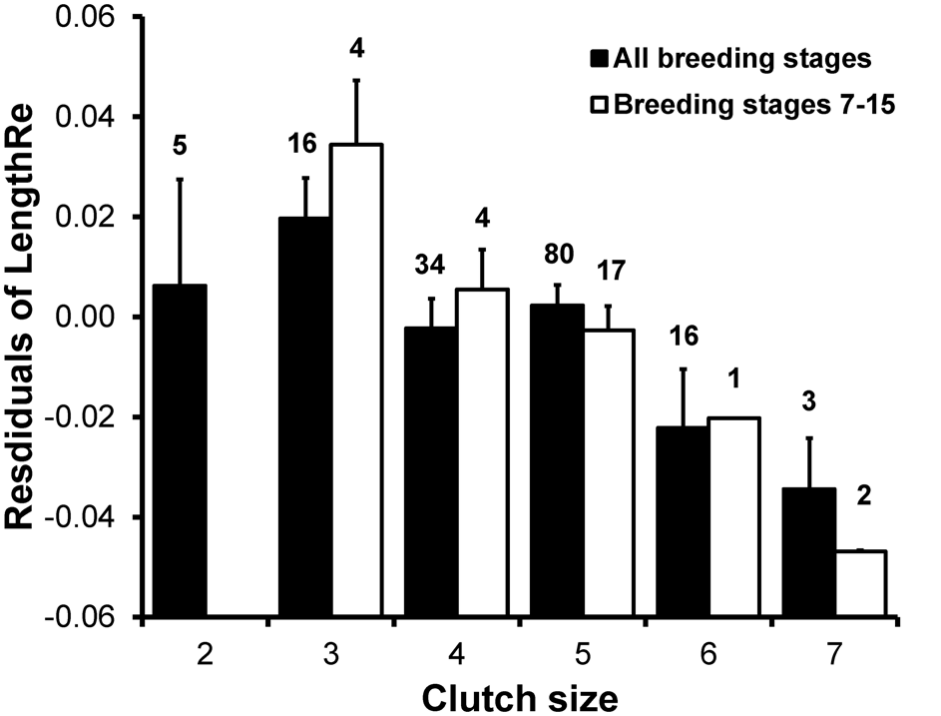
Mean (±SE) residual growing R_4_ length in relation to clutch size. Mean (±SE) residuals of LengthRe (see Methods) of females from models including breeding stage and date in relation to clutch size, for all breeding stages or for breeding stages ranging between 7 and 15 (see Results). Numbers above bars indicate sample sizes.

LengthRe was analyzed in relation to clutch size in a linear model where we also controlled for breeding stage and date. In females the analysis extended to the whole range of breeding stages showed a significant linear effect of clutch size (Table 5; Fig. 5 and 6), after excluding the non-significant quadratic term of clutch size and of breeding stage. Restricting the range of breeding stages disclosed a clear pattern of variation in the size of the effect on LengthRe (Fig. 5). The strongest, effects of breeding stage were consistently attained for ranges of breeding stages centered around 10–16 days after first egg laying and spanning over 9–22 days, i.e. for breeding stages ranging from the day of laying of the second egg, till breeding stage = 27, i.e. around the mid of the nestling period (Fig. 5). The largest effect size of breeding stage at feather removal was attained when the breeding stages in the interval (6,16) were considered in the analyses (Fig. 6).

**Table 5:** Length of the growing replacement feather in relation to clutch size.

		*T*	d.f.	*P*	Coefficient (±SE)
Females					
	Intercept				−0.101 (0.040)
	Clutch size	−2.68	150	0.008	8.4×10^−3^ (3.2×10^−3^)
	Breeding stage	−3.66	150	<0.001	1.1×10^−3^ (3.1×10^−4^)
	Feather removal date	4.02	150	<0.001	1.1×10^−3^ (2.6×10^−4^)
Males					
	Intercept				−0.161 (0.062)
	Clutch size	−0.18	151	0.857	7.8×10^−4^ (4.3×10^−3^)
	Breeding stage	−3.59	151	<0.001	1.4×10^−3^ (3.9×10^−4^)
	Breeding stage^2^	−2.64	151	0.009	−9.4×10^−5^ (3.4×10^−5^)
	Feather removal date	3.09	151	0.002	1.3×10^−3^ (4.3×10^−4^)

Linear model of the residuals of replacement R_4_ length relative to original R_4_ length on time since original feather removal (LengthRe in the text), on clutch size, breeding stage and date at original feather removal. For males, the second-order polynomial term of breeding stage is also included (see Results).

In the analysis of LengthRe on males, the second-order polynomial term of breeding stage was also included (see *Length of the replacement feather*). This analysis showed no significant association between LengthRe and clutch size of their social mate (Table 4; Fig. 5). This result held independently of the particular interval of breeding stages to which the analysis was restricted (*p* always>0.05) (Fig. 5).

## Discussion

We analyzed the trade-off between reproduction and feather growth in a small passerine bird which normally displays no temporal overlap between reproduction and molt, by monitoring the replacement of a tail feather that we had removed during the breeding season. In females, the width of the growth bars on the replacement feather decreased the later removal of the original feather was relative to the day of first clutch initiation, whereas this was not the case for males. In both sexes, the length of the growing replacement feather at any given time since removal of the original feather declined the later the original feather had been removed in an individual’s breeding cycle. Moreover, in females a negative relationship existed between GBW or length of the growing replacement feather and clutch size.

Physiological trade-offs under constraining ecological conditions are major determinants of life-histories because channeling of limiting resources into any demanding activity must occur to the detriment of allocation to other, competing activities [1], [2], [3], [4]. The present findings in combination with well-established information on the costs of reproduction and the sparse knowledge on the costs of feather production strongly and coherently support the idea that a physiological trade-off exists between reproduction and the ability to produce new feathers. The negative associations between GBW (females) or growing replacement feather length (both sexes) and breeding stage were more pronounced later in the first breeding attempt, suggesting that such physiological trade-offs are exacerbated as cumulative investment in breeding increases. The negative association between growing replacement feather length and clutch size was stronger after clutch initiation and for females with large clutches, and was absent in males, clearly hinting at a trade-off between allocation to egg vs feather production.

Barn swallows, like other Palearctic long-distance migratory birds, display a single annual molt of the flight feathers during wintering in sub-Saharan Africa [12]. In addition, they have an extremely long breeding season, with some pairs producing up to three broods from April to August [38], [56]. This study is therefore consistent with the hypothesis that temporal (and spatial) segregation between breeding and winter molt, which is thought to be a derived condition from the ancestral state of summer post-breeding molt for Palearctic species, may also be maintained because of a trade-off between reproduction and feather production [29], [30]. An additional specific aspect of our results that deserves attention is the significant increase that we observed in GBW with breeding stage of females but not males during the pre-laying period. Barn swallows may be regarded as income breeders. However, females gain mass during the pre-laying and early laying period, producing a hump-shaped pattern of temporal variation in individual body mass in the pre-laying till the egg hatching period (our unpublished results). This is likely to be an adaptation to sustain the cost of production of up to seven eggs (with a total mass of ca. 50% of female body mass), which are laid at one-day intervals, and perhaps also of incubation, which is performed by females alone (in the Western Palearctic) and entails a reduction of time devoted to foraging. Accumulation of mass before laying, which is functionally related to reproduction, may thus has the side-effect of promoting the speed of the replacement feathers whose growth is started just before clutch initiation.

There were obvious differences in variation of GBW and LengthRe according to breeding stage in the two sexes. The patterns we observed for females were consistent with our expectations, which hinged on the costs of egg production and of incubation (e.g. [57]). For males, equivocal predictions stemmed from lack of quantitative knowledge of the time and energy costs that males sustain for socio-sexual activities. The present results on variation in LengthRe of males suggest that feather production potential also declines after breeding stage −10, i.e. soon after arrival from migration and the start of socio-sexual activities.

In general, the proximate physiological mechanisms that produce the observed variation in replacement feather growth remain matter of speculation, because we lack information from experimental studies on the effect of change in hormonal profile on feather growth during the breeding season. High circulating levels of androgens and estrogens are known to impair feather molt [58], [59], while a raise in prolactin triggers onset of molt [60]. In males, inhibition of feather growth after the onset of laying by their mates may reflect such hormonal changes. Androgen levels of males are observed to peak around the onset of egg laying by their mates and to decrease thereafter [61]. Moreover, male prolactin increases from the pre-laying to the egg incubation phase, also in species where males do not participate in incubation, as is the case in Palearctic barn swallows [62]. In females, previous studies of field metabolic rates have shown that egg laying is not more costly than incubation or chick rearing [63], implying that we can rule out that changes in overall energy expenditure are the main cause of the observed changes in feather growth during breeding. However, the metabolic and hormonal changes that occur during the breeding cycle may have caused variation of replacement feather regrowth potential in relation to breeding stage. For instance, during the pre-laying period, female birds experience marked physiological changes, including a raise in the levels of steroid hormones (mainly estrogens) and gonadotropins, whose levels rapidly dwindle with the onset of incubation (see [64]). Prolactin levels are low during this phase, but rapidly increase post-laying with the onset of incubation [65], [66]. Finally, corticosterone may also be involved in the control of replacement feather growth. High corticosterone levels are known to reduce feather growth and alter feather structure and melanin deposition[67] [68]. Increase in corticosterone levels during breeding [69] may thus be responsible for the observed patterns of replacement feather growth.

Interestingly, GBW and length of the growing replacement feather were positively but only weakly correlated, particularly after controlling for the spurious effects of breeding stage, date and clutch size. This was the case despite these two measures of feather production ability may be viewed as inherently correlated, because the former should partly determine the latter. In females, however, GBW reached a peak in individuals whose original feather had been plucked around clutch initiation while growing replacement feather length declined linearly with breeding stage. In addition, in males GBW was unrelated, whereas growing replacement feather length declined, with breeding stage. Uncoupling of these variables may result from latency in the start of ReR_4_ growth being unrelated to subsequent rate of growth (and thus GBW). This suggests that in males breeding stage mainly affected the time ReR_4_ took to start growing.

Clearly, more experimental work is thus needed to identify the proximate physiological mechanisms that affect feather production after the onset of the core breeding period and also on the mechanisms that uncouple re-activation of the feather follicle to start producing a new feather from later rate of feather growth as reflected by GBW.

The present results may have a bearing for the interpretation of the evolution and maintenance of temporal separation between molt and other activities, such as breeding. The few previous studies of the molt-breeding trade-off in species with facultative overlap of these activities have provided different forms of evidence for reciprocally constraining relationships [15], [16], [17], [18], [19]. Temporal separation between molt and breeding is believed to have evolved from an ancestral condition of overlap between the two activities in boreal species that winter south of the Sahara [40], [41]. These long-distance migratory species with winter molt affords an opportunity to study selection for maintenance of temporal segregation. The present results suggest that trade-offs between breeding and feather replacement costs tend to maintain the current obligate segregation of molt and breeding in species that undergo a complete molt in their sub-Saharan wintering quarters.

In conclusion, we have shown that the ability of barn swallows to produce new feathers declines as they enter their core breeding period, suggesting a physiological trade-off caused by limiting availability of resources for breeding and feather biosynthesis and possibly, proximately mediated by changes in circulating steroid hormones or prolactin. This trade-off was most apparent in females, which also showed a marked decline in their ability to produce new feathers particularly after clutch completion and an intense reproductive effort as reflected by large clutch size. Hence, this study suggests that temporal segregation between winter molt and breeding in European long-distance migratory birds could be maintained by selection for avoidance of a trade-off between feather biosynthesis and breeding. In addition, it supports the use of ptilochronology as a powerful, though largely under-exploited, tool to address a number of questions on the ecology and evolution of life-histories in birds.

## Supporting Information

Supporting Information S1 **Additional methodological information.**
(DOC)
