# Supplementary material for: A Trade-Off between Reproduction and Feather Growth in the Barn Swallow (Hirundo rustica)
Source: PLoS One. 2014 May 14;9(5):e96428. doi: 10.1371/journal.pone.0096428 (PMC4020794; doi:10.1371/journal.pone.0096428)
Supplement: Supporting Information S1 — Additional methodological information. (DOC) [file pone.0096428.s001.doc]

**Supporting Information (SI)**

**Additional methodological information**

**SI.1.** Date of clutch initiation was recorded directly or inferred retrospectively assuming that the 2-7 eggs in a clutch are laid at one-day intervals and that incubation lasts 14 days starting on the day of laying of the penultimate egg, and also by estimating age of newly hatched chicks, according to well established protocols (see [1], [2]).

**SI.2.** Barn Swallows can be efficiently capture at breeding colonies by putting mist-nests at any entrance of the cattle-sheds or of the other buildings where they nest before dawn. Timing of recapture to measure the length of the growing replacement feather before it completed its growth was chosen based on pilot observations to achieve four main endpoints: 1) allowing the replacement feather to grow to a length sufficient for measuring growth bar width on 9 growth bands at the distal end of the feather (see main text and below); 2) measuring the growing replacement feather before it had completed growth in most cases, in order to analyse not only growth bar width but also actual growth of the feather, as this depends both the rate at which the replacement feather grows and the latency in start of growth of the replacement feather after the original feather is removed; 3) in a subset of birds (ca. 10%), sampling fully regrown replacement feather to obtain an asymptotic estimate of final replacement R_4_ length and to compare the final length of the replacement feather with the length of the original feather of the same individual.

**SI.3.** The conditions under which GBW was measured are those under which the bars are most visible, according to our experience with barn swallow feathers. In most cases, the light source was approximately located 60° above the plane of the vane, laterally to the feather and on the same side of the feather where the observer was located. However, in some cases the light source was opportunistically shifted vertically (i.e. changing the inclination of the incident light) or parallel to the rachis to make the growth bars appear more clear. In all cases growth bars were measured under the conditions where they appeared more clearly to the observer.

**SI.4.** Piecewise regression is a form of regression that allows multiple linear models to be fit for different ranges of the independent variable. The breakpoint(s) between the two (or more) ranges of the independent variable may or may not be known in advance. As the latter was the case in the present study, we identified the approximate breeding stage at which any discontinuity appeared to be most apparent using LOESS regression, as suggested by Ryan & Porth (2007), in order to identify tentative starting values to include in the piecewise regression analysis. Polynomial regression analysis was intended for confirmatory purposes or to obtain an estimate of any deviation from a linear variation and of the position of any maxima/minima in cases when piecewise regression models failed to converge.

**SI.5.** In exploratory analyses of the association between residual ReR_4_ length or GBW and clutch size we identified discrete intervals of breeding stages when the OrR_4_ had been plucked differing in median date and in the time span they encompassed. For the analyses of residual ReR_4_ length we used all the possible intervals lasting between 9 and 51 days (i.e. the maximum span of breeding stage between -20 and +30) and differing in duration by multiple of 3 days. Thus, the analyses of the association between residual ReR_4_ length and clutch size were done over the entire span of breeding stages encompassed by the study (i.e., [-20,30]), then in the two intervals (breeding stage: [-20,27]; [-17,30]; including the extremes) spanning 48 days intervals, in the 3 intervals spanning 45 days ([-20,24]; [-17,27]; [-14,30]), and so forth for the intervals of shorter duration, up to a minimum duration of 9 days. A similar approach was used for the association between GBW and clutch size but using all the intervals spanning in amplitude between 21 and 51 days and differing in duration by multiple of 3 days. Thus, the analyses were done on the following breeding stage intervals (including the extremes): ([-20,30]; [-10,30]; [0,30]; [10,30]; [-20,20]; [-20,10]; [-20,0]; [-10,20]; [-10,10]; [0,20]. The coarser grain in the analysis of GBW was adopted because the strength of the association between GBW and clutch size was obviously less differentiated among breeding stages at OrR_4_ plucking.

References

1. Ambrosini R., Bolzern AM, Canova L, Arieni S, Møller AP, et al. (2002) The distribution and colony size of barn swallow in relation to agricultural land use. J Appl Ecol 39: 524-534.

2. Ambrosini R., Saino N. (2010) Environmental effects at two nested spatial scales on habitat choice and breeding performance of barn swallow. Evol Ecol 24: 491-508.

3. Ryan SE, Porth LS (2007) A Tutorial on the Piecewise Regression Approach Applied to Bedload Transport Data. Gen. Tech. Rep. RMRS-GTR-189. Fort Collins, CO: U.S. Department of Agriculture, Forest Service, Rocky Mountain Research Station. 41 p.
